# Venous thromboembolism risk assessment of surgical patients in Southwest China using real-world data: establishment and evaluation of an improved venous thromboembolism risk model

**DOI:** 10.1186/s12911-022-01795-9

**Published:** 2022-03-04

**Authors:** Peng Wang, Yao Wang, Zhaoying Yuan, Fei Wang, Hongqian Wang, Ying Li, Chengliang Wang, Linfeng Li

**Affiliations:** 1 College of Computer Science, Chongqing University, Chongqing, China; 2 Yidu Cloud Technology Inc, Beijing, China; 3 Center for Applied Statistics and School of Statistics, Renmin University of China, Beijing, China; 4 Medical Big Data Center of Southwest Hospital, Chongqing, China

**Keywords:** Venous thromboembolism, Risk assessment model, Caprini, Surgical patients, Machine learning

## Abstract

**Background:**

Venous thromboembolism (VTE) risk assessment in surgical patients is important for the appropriate diagnosis and treatment of patients. The commonly used Caprini model is limited by its inadequate ability to discriminate between risk stratums on the surgical population in southwest China and lengthy risk factors. The purpose of this study was to establish an improved VTE risk assessment model that is accurate and simple.

**Methods:**

This study is based on the clinical data from 81,505 surgical patients hospitalized in the Southwest Hospital of China between January 1, 2019 and June 18, 2021. Among the population, 559 patients developed VTE. An improved VTE risk assessment model, SW-model, was established through Logistic Regression, with comparisons to both Caprini and Random Forest.

**Results:**

The SW-model incorporated eight risk factors. The area under the curve (AUC) of SW-model (0.807 [0.758, 0.853], 0.804 [0.765, 0.840]), are significantly superior (*p* = 0.001 and *p* = 0.044) to those of the Caprini (0.705 [0.652, 0.757], 0.758 [0.719, 0795]) on two test sets, but inferior (*p* < 0.001 and *p* = 0.002) to Random Forest (0.854 [0.814, 0.890], 0.839 [0.806, 0.868]). In decision curve analysis, within threshold range from 0.015 to 0.04, the DCA curves of the SW-model are superior to Caprini and two default strategies.

**Conclusions:**

The SW-model demonstrated a higher discriminative capability to distinguish VTE positive in surgical patients compared with the Caprini model. Compared to Random Forest, Logistic Regression based SW-model provided interpretability which is essential in guarantee the procedure of risk assessment transparent to clinicians.

**Supplementary Information:**

The online version contains supplementary material available at 10.1186/s12911-022-01795-9.

## Background

Venous thromboembolism (VTE) is a venous occlusive disease characterized by abnormal coagulation of blood in the vein [1]. VTE can affect veins in various parts of the body. It is a common preventable disease with a high recurrence rate, mainly including deep venous thrombosis (DVT) and pulmonary thromboembolism (PTE) [2, 3].

The incidence of VTE of the general population is 0.1–0.2% in Western countries [4] and 0.0088–0.013% in Asian countries [5, 6]. The incidence rate is 1.24%, 0.67%, and 0.05% in orthopedic surgery patients, cancer surgery patients, and benign surgery patients, respectively [7]. A multi-center study conducted in China showed that the annual mortality rate of hospitalized VTE patients increased from 2.1% to 4.7% between 2007 and 2016 [8]. Moreover, the occurrence of VTE significantly adds to the economic burden of hospitalized patients. According to a survey conducted in the United States, the direct medical cost of VTE was even higher than that of stroke [9]. According to VTE management guidelines published by the American Society of Hematology in 2018 [10] and the European Society of Cardiology in 2019 [11], appropriate diagnostic strategies for VTE are based on assessment of the pretest probability(PTP) for individual patients, and the ability of diagnostic tests, such as D-dimer and ultrasound [10, 11], is not only influenced by test accuracy characteristics but also influenced by PTP. Therefore, it is necessary to conduct VTE risk assessment for accurate PTP prediction to formulate appropriate diagnostic strategies and reduce VTE morbidity, mortality and medical expenses, as well as improve patient prognosis, and improve the quality of life [12].

Validation studies and preliminary practical experience have shown that the Caprini Thrombosis Risk Assessment Scale is an effective and feasible VTE RAM for postoperative patients [13]. This risk assessment scale was published in 1991 [14] and has been revised several times since then [15, 16]. The Caprini scale comprehensively evaluates the VTE risk factors in surgical patients. However, for the hospitalized Asian population, use of the Caprini scale has certain limitations. The incidence of VTE in the Asian population is significantly lower than that in the Western population. Moreover, in Asia, most surgical patients are middle-aged or elderly, and the surgery time is usually longer than 45 min; therefore, the use of Caprini score stratifies most Asian surgical patients to the high risk partition and overestimates the VTE risk, which leads to unnecessary anticoagulation therapy and increases the bleeding risk and economic burden on the patients [17, 18].

Besides risk assessment scores like Caprini, recent studies had applied machine learning methods, including Supporting Vector [19] and Random Forest [20], to VTE risk stratification. Artificial Neural Network was also found effective to analysis risk factors [21]. Ensemble learning algorithm was further applied to improve discrimination and calibration [22]. It was suggested that these machine learning-based models show more elaborated and accurate risk prediction than traditional scores [22].

Despite advantages, there is hardly any VTE RAM built by machine learning approaches widely used in clinical practice. The main obstacle is the black box nature of many machine learning algorithms [23]. Without interpretability, the inference result is not transparent to clinicians, thus the reliability cannot be trusted. In contrast, the risk assessment result of Caprini could be directly attribute to several risk factors. Such transparency makes Caprini easy-to-understand.

We collected the medical records of surgical patients from Southwest Hospital, a comprehensive tertiary hospital in Southwestern China, between January 1, 2019 and June 18, 2021 (hereinafter referred to as the study dataset). A total of 559 patients developed VTE, with an incidence rate of 0.686%. It was found that 86% of the surgical patients were stratified into medium, high or highest risk by Caprini RAM. This indicates that the Caprini RAM seriously overestimated the VTE risk, which echoes the limitations discussed in previous works [24].

To address the limitations of Caprini and keep the interpretability, this paper developed an improved version of the VTE RAM using Logistic Regression from surgical patients in southwest China, named as SW-model. The SW-model and benchmark models are evaluated on both retrospective and prospective test datasets. It is proved the SW-model had a significantly better discriminative ability than Caprini in both test datasets, while providing interpretable results compared to Random Forest.

## Methods

### Study population

This study included surgical patients discharged from the Southwest Hospital between January 1, 2019 and June 18, 2021. We included patients aged ≥ 18 years who were hospitalized for longer than 2 days and discharged from the designated departments. We excluded patients who were diagnosed with DVT or PTE at the time of admission. A total of 81,505 patients were selected as study population.

The study population is spitted into training dataset, retrospective test dataset and prospective test dataset. Training dataset comprises patients discharged from 2019 to 2020, except those 20% who were randomly selected into retrospective test dataset. The prospective test dataset comprises patients who were discharged in 2021.

**Fig. 1:**
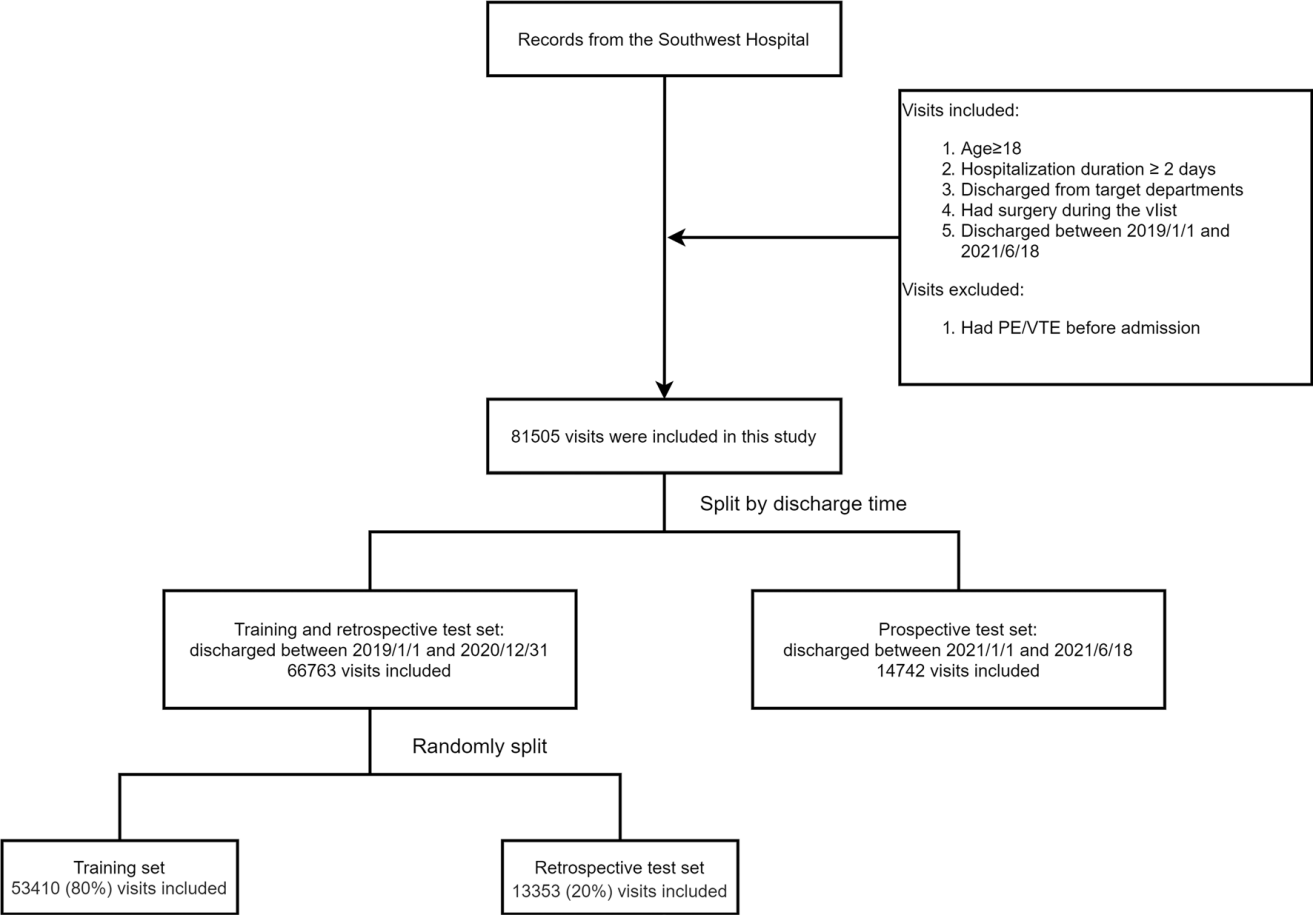
Flow chart of study population construction and splitting into training, test datasets

The flow of preparing study population and splitting into training and test datasets are illustrated in Fig. 1.

### Outcomes

The development of fresh VTE during the hospital stay was considered as a clinical observation event. Based on the diagnostic rules from VTE disease management guidelines [10, 11], a positive event was defined as below:

1. The ICD-10 code of discharge diagnosis contains DVT or PTE, or
2. Findings of the upper or lower extremity blood vessel ultrasound or CT examination suggestive of DVT, or
3. Findings of CT angiography of pulmonary artery or lung perfusion scan suggestive of PTE.

The detailed implementation of the definition, including which range of ICD-10 codes are considered to be VTE, PTE and what pattern suggest DVT or PTE in exam report, is described in Additional file 1: Table S1.

### Risk factor extraction

We developed a specialized program to extract information from the electronic medical record system, such as hospital information system (HIS), laboratory information system(LIS), radiology information system(RIS), surgery and anesthesia information system, etc. The risk factor extraction process involved extraction from structured and unstructured information. Structured information refers to data stored in structured form in existing system, such as age at the time of hospital visit and abnormal test results. Unstructured information refers to text of electronic medical records, which require semantic analysis and medical logical reasoning to extract risk factors (e.g. presence of varicose veins and history of arthroscopic surgeries).

To extract risk factors from unstructured information, a data processing pipeline is used. The first phase is data preparation, where the raw medical records from various system are aggregated into visit level. The next one is entity recognition, where diagnosis, symptom, treatment activity could be extracted. The third phase is entity normalization, which map different expressions of same entity into the standard code. After normalized entity, the risk factors could be determined. This pipeline is supported by data process and application platform (DPAP) at the Southwest Hospital. In one of our previous work [25], the details of pipeline are described.

### Feature engineering

The feature engineering includes construction of full feature set, discretize continuous feature into categorical feature, and feature selection. The full feature set contains all risk factors from Caprini RAM, and extra risk factors from previous works. The full feature set is described in Additional file 1: Table S2. Continuous features were discretized into categorical ones using algorithms based on Chi-square test [26] and Kolmogorov–Smirnov test [27]. To discretize continuous features, including age and surgery duration, the optimal cut-off thresholds were determined by ten-fold cross validation on training set only, refer to Additional file 1: Figure S1. The univariate odd ratio (OR) of each feature was tested by two-sided Z test. The significance of test is used to select candidate features from full feature set.

### Models and evaluation

The 2005 version of Caprini RAM is selected as benchmark, the risk factors and scores of which are listed in Additional file 1: Table S3. According to the previous study [15], a total risk score greater or equal to 5 is highest risk stratum, risk score between 3 to 4 is high risk, risk score 2 is medium, the other is low risk.

The improved RAM is developed using machine learning methodology. Specifically, we compared Logistic Regression and Random Forest in building RAMs. In Logistic Regression, step-wise feature selection is applied and the model is fitted by max likelihood estimation. In Random Forest, the number of trees is set to 500, the maximum depth is set to 8.

The discriminative capabilities of models were measured by area under ROC curve (AUC), on both retrospective test dataset and prospective test dataset. The sensitivity, specificity, Youden's index [28], positive predictive value (PPV) and negative predictive value (NPV) were reported. Delong test [29] was used to compare differences in AUC.

Considering in clinical application risk stratums are more commonly used than risk value, patients were stratified into different stratums based on model output. For Caprini, the stratifying strategy had been stated in the beginning of this section. For improved models, the VTE risk of patients could be stratified into four stratums using the threshold-moving method. The goal of stratifying strategy is making the VTE incidence rate in medium risk stratum similar to the average level of study population, while high and low stratum significant than medium level.

To compare the clinical benefits among models, decision curve analysis (DCA) [30, 31]was used.

All statistical analyses were performed using python-based scientific computing package, including scipy [32], numpy [33], and scikit-learn [34], statsmodels [35]. For all hypothesis tests, α = 0.05 is selected as the significance level.

## Results

### Patient characteristics

The distributions of important features, which are selected according to previous studies [36] and expert opinions, are shown in Table 1. The distributions of full features are listed in Additional file 1: Table S2.

**Table 1:** Comparison of the characteristics of study participants on training, retrospective test and prospective test dataset

	Train set (2019–2020)	Retrospective test set (2019–2020)	*p* value between training and retrospective test set	Prospective test set (2021)	*p* value between training and prospective test set
Number of visits	53,410	13,353		14,742	
Venous thromboembolism	339 (0.63%)	85 (0.64%)	0.981	135 (0.92%)	< 0.001
Age: 18–40 years	14,332 (26.83%)	3571 (26.74%)	0.832	3571 (24.22%)	< 0.001
Age: 41–60 years	23,836 (44.63%)	5924 (44.36%)	0.584	6902 (46.82%)	< 0.001
Age: 61–75 years	12,301 (23.03%)	3098 (23.2%)	0.677	3379 (22.92%)	0.778
Age: 75 + years	2941 (5.51%)	760 (5.69%)	0.403	890 (6.04%)	0.013
Gender: male	23,412 (43.83%)	5890 (44.11%)	0.566	6622 (44.92%)	0.018
Gender: female	29,998 (56.17%)	7463 (55.89%)	0.567	8120 (55.08%)	0.020
BMI > 25 kg/m^2^	17,878 (33.47%)	4482 (33.57%)	0.840	5233 (35.5%)	< 0.001
Bedridden status	3454 (6.47%)	891 (6.67%)	0.388	1390 (9.43%)	< 0.001
History of DVT or PE	86 (0.16%)	33 (0.25%)	0.036	40 (0.27%)	0.006
Malignancy	15,846 (29.67%)	3867 (28.96%)	0.109	4768 (32.34%)	< 0.001
Abnormal platelet counts	1276 (2.39%)	311 (2.33%)	0.684	337 (2.29%)	0.466
Abnormal carcinoembryonic antigen levels	1065 (1.99%)	257 (1.92%)	0.607	266 (1.8%)	0.141
Abnormal triglyceride levels	1745 (3.27%)	434 (3.25%)	0.921	617 (4.19%)	< 0.001
Abnormal hemoglobin levels	1832 (3.43%)	460 (3.44%)	0.933	530 (3.6%)	0.332
Major surgery	39,463 (73.89%)	9865 (73.88%)	0.984	11,412 (77.41%)	< 0.001

Bedridden status refers to the patient's bedridden status at admission or doctor's order of bed rest during hospitalization. Abnormal platelet count refers to platelet count > 350 × 10^9^/L in the month before hospitalization. Abnormal carcinoembryonic antigen levels refer to levels > 5 ng/mL in the month before hospitalization. Abnormal triglyceride levels refer to levels > 1.7 mmol in the month before hospitalization. Abnormal hemoglobin levels refer to levels > 100 G/L in the month before hospitalization

Table 1 demonstrated that VTE incidence rate and most important features share similar distributions between the training and retrospective test datasets, except ‘History of VTE or DVT’.

The distributions of some features between training and prospective test set are significant different: the VTE incidence rate was significantly higher on the prospective test set compared with the training set (0.92% and 0.63%, respectively; *p* < 0.001) Patients in the prospective test set were older than those in the training set; specifically, the proportion of 41–60-year-old patients was significantly higher and the proportion of 18–40-year-old group was significantly lower than training set. Besides, the proportions of patients with BMI greater than 25, patients with bedridden status, patients with malignancy, patients with abnormal triglyceride levels, patients with surgery longer than 45 min were significantly higher than that in the training set. Notably, the differences between train set and prospective test set shown in Table 1 are a result of changes in the real-world data, not selection bias.

### Model development

Patients were divided into age groups (18–40 years; 41–60 years; 61–75 years; and > 75 years) using the same thresholds as the Caprini model. The threshold to distinguish major and minor surgeries was adjusted to 180 min according to univariate and multivariate AUC of ten-fold cross validation.

The SW-model is derived from training dataset using logistic regression. The coefficients of each feature is reported in Table 2.

**Table 2:** Coefficients and adjusted odds ratios of each feature in SW-model

Feature name	Coefficients (*p* value)	Adjusted odds ratio (95% confidence interval)
History of DVT or PE	3.575 (*p* < 0.001)	35.701 ([18.266,69.779])
Septicemia	1.737 (*p* = 0.008)	5.681 ([1.589,20.312])
Serious lung disease	1.599 (*p* < 0.001)	4.946 ([3.296,7.422])
Bedridden	1.277 (*p* < 0.001)	3.585 ([2.744,4.685])
Fluid blood in operation	1.187 (*p* < 0.001)	3.277 ([2.484,4.324])
Operation duration: > 180 min	1.152 (*p* < 0.001)	3.164 ([2.480,4.036])
Age: > 75 years	0.913 (*p* < 0.001)	2.493 ([1.751,3.548])
Abnormal serum homocysteine levels	0.883 (*p* < 0.001)	2.418 ([1.687,3.464])

**Fig. 2:**
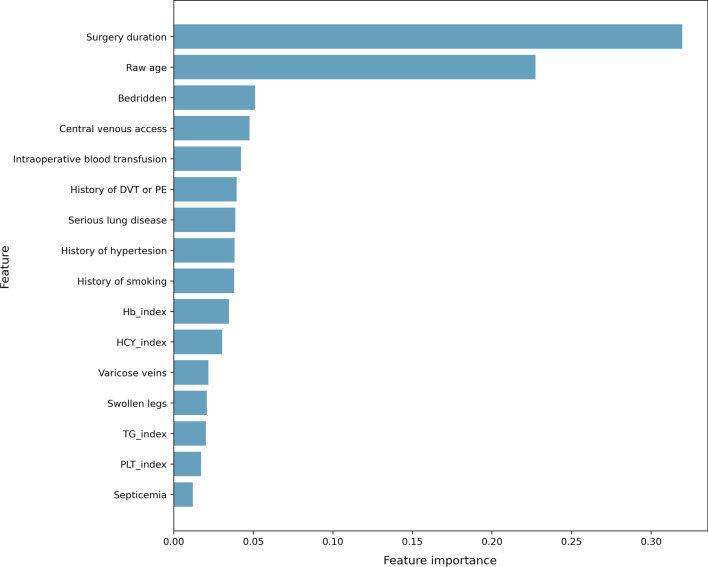
Feature Importance of Random Forest

In addition to SW-model, another benchmark model is developed by Random Forest. The feature importance of Random Forest model is reported in Fig. 2.

### Model evaluation

**Fig. 3:**
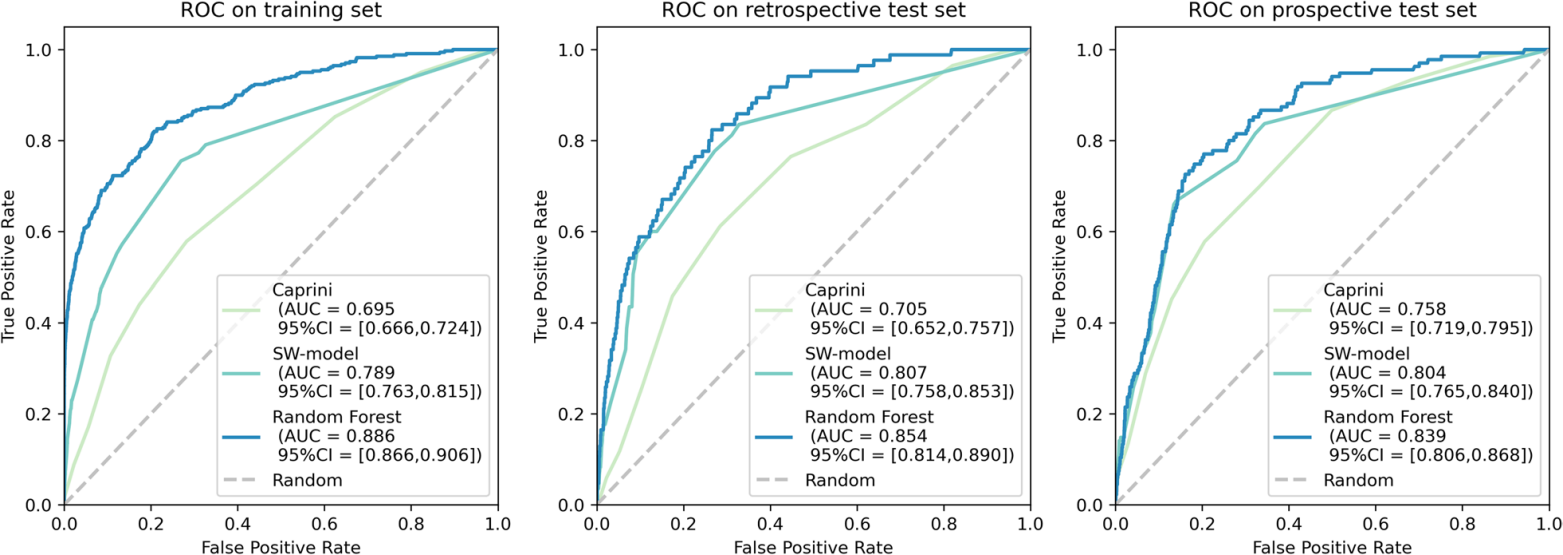
ROC and AUC (95% CI ) of the SW-model and Caprini model in the test set. Notes: *p* value between Caprini and SW-model: 0.001*** on retrospective test set, 0.044* on prospective test set. *p* value between Random Forest and SW-model: < 0.001*** on retrospective test set, 0.002** on prospective test set. *p* value between retrospective and prospective test set: Caprini 0.116, SW-model 0.934, Random Forest 0.558

The AUC values for Caprini model, the SW-model and Random Forest model in the training set, retrospective test set and prospective test dataset are shown in Fig. 3. On both retrospective and prospective test set, SW-model is significantly better than Caprini model and significantly inferior to Random Forest. The AUCs of all models are not significant different between retrospective and prospective test datasets.

The sensitivity, specificity, PPV and NPV is compared among models in Table 3 in three different scenarios: “high sensitivity scenario” where the thresholds of each model was selected to achieve at least 80% sensitivity, “high specificity scenario” the thresholds of each model was selected to achieve at least 90% specificity, and “optimal Youden's index scenario”.

**Table 3:** Comparison of sensitivity, specificity, Youden's index, PPV and NPV on prospective test set

Scenario	Model	Threshold	Sensitivity	Specificity	Youden’s index	PPV	NPV
High sensitivity	Caprini	5	0.867	0.501	0.368	0.016	0.998
	SW	0.006	0.837	0.656	0.493	0.022	0.998
	RF	0.005	0.867	0.665	0.532	0.023	0.998
Optimal Youden's index	Caprini	7	0.578	0.794	0.372	0.025	0.995
	SW	0.008	0.667	0.861	0.527	0.042	0.996
	RF	0.009	0.770	0.796	0.567	0.034	0.997
High specificity	Caprini	9	0.289	0.930	0.219	0.037	0.993
	SW	0.020	0.356	0.928	0.283	0.043	0.994
	RF	0.016	0.363	0.931	0.294	0.046	0.994

Threshold, the discriminative threshold above which the patients is predicted to be “positive”; SW, SW-model; RF, random forest

**Fig. 4:**
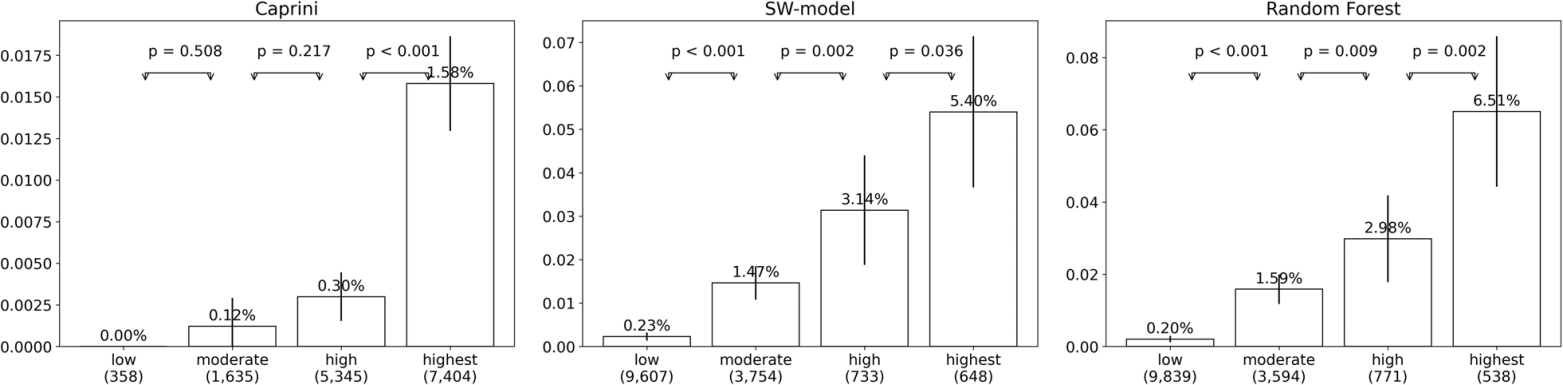
VTE incidence rate and number of patients in different risk stratums on prospective test dataset. Notes: The number in brackets, e.g. ‘358’ in “low (358)” in left sub-graph, represent number of patients who are classified into the stratum

To stratify patients into different risk stratums, for SW-model the predicted probability of 0–0.005, 0.005–0.01, 0.01–0.025, and > 0.025 were selected to be ranges for low, medium, high, and highest risks, respectively. The thresholds of Random Forest, were 0.005, 0.014 and 0.025 to stratify patients into different VTE risk stratums. To validate the ability of stratifying patients into different risk stratums, in Fig. 4, the number of patients, the incidence rate of each risk stratum, and inter-stratum differences in the prospective test set were compared among models.

**Fig. 5:**
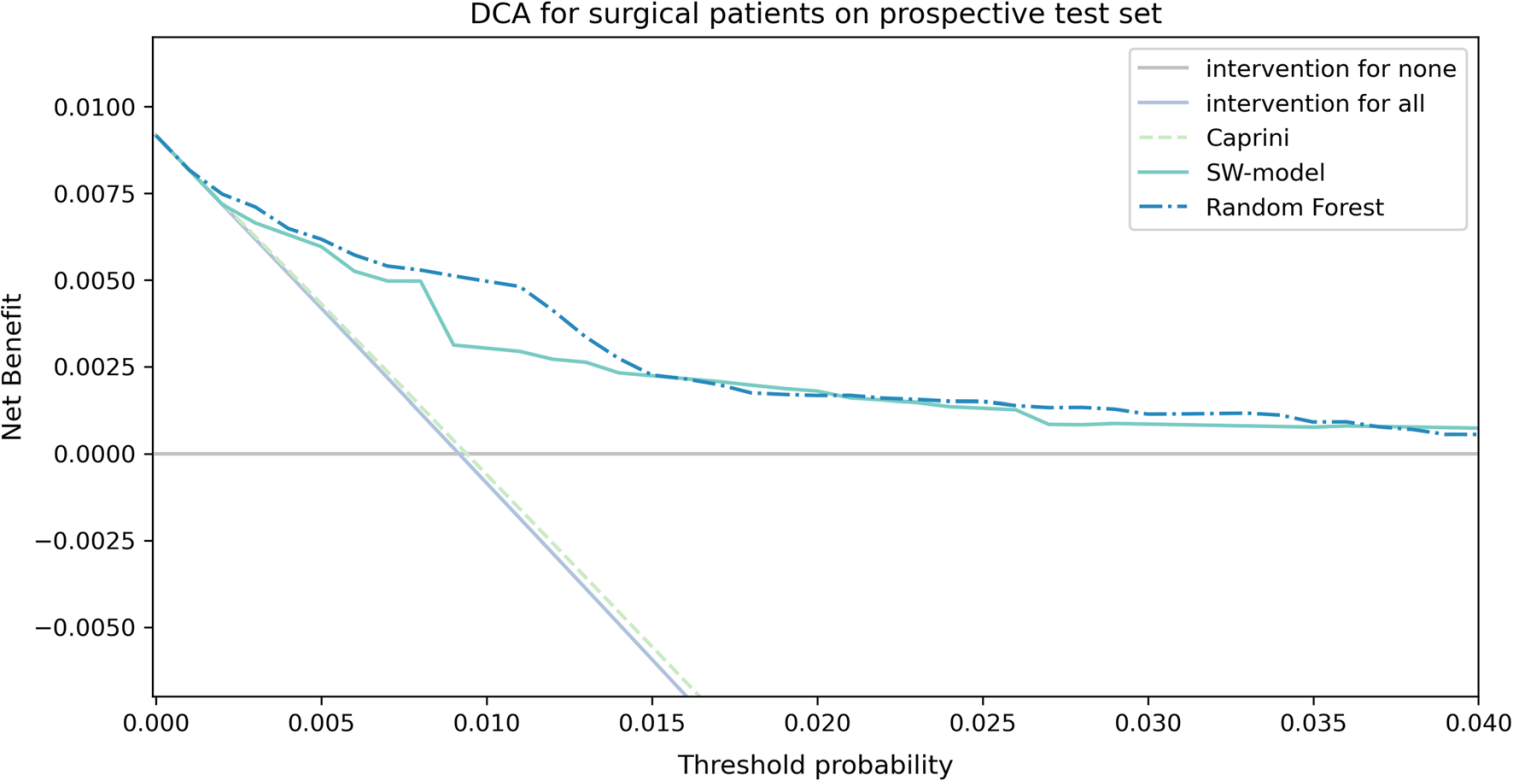
Decision curve analysis for the SW-model and Caprini model

To evaluate the decision benefits of to develop strategies to prevent VTE or PTE in the clinical setting, DCA curves were produced for the models and two default strategies, referring to treating none or all of patients (Fig. 5). Within threshold range from 0.015 to 0.04, the DCA curves of the SW-model and Random Forest are superior to Caprini and those two default strategies.

## Discussion

Based on real-world data of surgical patients collected between January 1, 2019 and June 18, 2021 at the Southwest Hospital, this study established an improved VTE risk assessment model that demonstrated better classification capability than the Caprini model, and more practical for clinical to use other machine learning algorithms such as Random Forest.

### Influence of COVID-19

The time span of study dataset covered the pandemic period of COVID-19. It has been reported that pro-thrombotic derangement of the hemostatic system is a prominent feature among clinical manifestations of COVID-19 [37, 38]. Therefore, the incidence rate of VTE in our study dataset may be influenced by COVID-19.

However, the influence of COVID-19 on VTE incidence rate of our studied population is indirect rather than direct. On one hand, there is no COVID-19 patient in the study population, because in China all COVID-19 patients were treated in designated hospitals while Southwest hospital was not among the designated hospitals. On the other hand, there were huge indirect impacts on the prevalence of VTE caused by COVID-19. During the lockdown periods in early 2020, patients stopped visiting hospitals in fear of being infected, except those with life-threatening conditions. Thus, the patients after 2020 was more serious than those in 2019, leading to more prevalence of in-hospital VTE. In Table 1 and Additional file 1: Table S2, the distribution of features echoed such trend.

As the COVID-19 continues in 2021, its indirect impact on in-hospital VTE incidence rate continued; but it is probably not the only reason. According to Table 1, surgical patients were older in year 2021 than those in year 2019 to 2020, which could also be attributed to the aging of Chinese society.

### Risk factors

The risk factors adopted in the SW-model (Table 2) were with those from previous studies. Among the eight risk factors that were included in the model, sepsis, severe lung diseases, VTE history, and serum homocysteine level are also included in the Caprini model. The characteristic of age retained the 75-year-old cut-off point; surgery length adopted the more reasonable cut-off point of 180 min. Two new factors of bed rest during hospitalization and blood transfusion during surgery were included. Regarding the four risk factors in common with the Caprini model, a number of studies [39–41] have confirmed that among surgical patients, those with severe chronic obstructive pulmonary disease have a higher risk of VTE. Data from Africa [42] and the United States [43] showed that sepsis is a risk factor of VTE. A case–control study [44] showed that moderately elevated serum homocysteine level is an independent risk factor for VTE. Moreover, the ROC slope for VTE history was relatively steep for the SW-model, which is consistent with a number of previous studies [45–47] that suggested that VTE history is one of the strongest risk factors for fresh VTE in the general population. A multi-center retrospective cohort study conducted in the United States [48] showed a direct relationship between duration of surgery and VTE risk, and recommended the use of quintiles for risk assessment. This study obtained the optimal cut-off point of 180 min using the feature binning technique that is more suitable for surgical conditions in China. The improved model also included age as a risk factor of VTE, while the scoring weights of different age groups were different from those of the Caprini model [15]. In particular, for patients aged 41–74 years, the Caprini model assigns an increased risk of VTE, while the model in this study does not.

For factors not included in the Caprini model, we found support from the results of previous studies. A 2018 study [36] reported that blood transfusion during the perioperative period significantly increased the VTE incidence, which is consistent with the impact of preoperative and intraoperative blood transfusions on VTE risk in this study. Regarding bed rest, previous pathological studies [49, 50] showed that bed rest can lead to venous stasis and increased VTE risk. A meta-analysis [38] also confirmed that bed rest increases the VTE risk in medical patients.

### Strengths and weakness of SW-model

First, the discriminative capability of SW-model, measured by AUC, was significantly improved from Caprini in both retrospective (*p* = 0.001) and prospective (*p* = 0.044) test datasets according to Fig. 3. Additionally, comparison of AUC between the training and each test dataset did not reveal any significant difference (*p* = 0.520, *p* = 0.513), indicating that the SW-model had good external validity. Regarding specificity, sensitivity and other metrics in Table 3, SW-model outperformed Caprini in most cases on prospective test set, except the highest sensitivity of SW-model is lower than Caprini. The difference is in align with the top-right part of ROC curve in the right sub graph in Fig. 3. The SW-model could identify 83% patients in risk of developing VTE, with higher PPV (less false alarms) than Caprini, but at cost of the other risk patients. To clinical applications, it is important to leverage SW-model’s specificity to address the challenge that Caprini stratifies most surgical patients to the high and highest risk stratum, leading to unnecessary anticoagulation therapy and increases the bleeding risk and economic burden on the patients.

To compare the AUC, sensitivity, specificity and other metrics between SW-model and Random Forest, it is obvious Random Forest is superior to SW-model. Considering the strength of tree-based algorithm is modelling non-linear relationship, the result implies that non-linear relationship existing between risk factors and VTE incidences.

Second, with better discriminative capability, SW-model could stratify patients into different risk stratums more accurate than Caprini. As Fig. 4 demonstrated, differences of VTE incidence rate among the low, medium and high-risk stratums by Caprini were not significant, while the highest risk stratum consists of more than half patients. That is to say, the clinicians get “highest risk” alarms on more than half patients, which causes unnecessary burden. In contrast, there are significant differences among the four risk stratums by the SW-model, and the proportion of patients in highest and high risk stratums reduced to reasonable level (4.4% and 5.0%). This finding indicate SW-model can identify the small proportion (< 10%) of patients who are extremely prone to VTE who will benefit from interventions to reduce the VTE risk.

Third, SW-model provided simplicity and interpretability. On simplicity, the SW-model can predict the VTE risk using only eight parameters that are easily available in routine clinical practice, which simplifies the complexity of clinical use of the Caprini RAM. The 2005 version of the Caprini RAM includes almost 40 risk factors from multiple information systems, such as medical history, diagnosis and treatment records, examination records, doctor’s orders, and surgery records. Obtaining such information requires significant more time and efforts than SW-model even automatic information extraction is deployed. On interpretability, SW-model, which build from logistic regression, is interpretable by nature. Such interpretability provides more transparency than Random Forest and other potential machine learning methods in clinical use. It is not easy, if possible, to understand how the 500 trees work together to produce a slight better result. Therefore, although the AUC of Random Forest outperformed SW-model (0.839 vs. 0.804, *p* = 0.002), the SW-model is proposed for clinical use.

Finally, the net benefits of SW-model outperformed Caprini. As in Fig. 5, The DCA curve of the Caprini model almost completely overlapped with that of the treatment-for-all strategy, because Caprini stratify 86.47% of the surgical patients into the high-risk partition and the highest-risk partition but the VTE incidence rate in the Caprini high-risk and highest-risk stratum were 0.30% and 1.58% respectively. If the Caprini model were used to design thrombosis prevention strategies, the resulting treatment strategy is close to the treatment-for-all strategy. Therefore, large-scale treatment of the Caprini model will be associated with unnecessary costs. In contrast, the SW-model can balance the risks of thrombosis formation and excessive anticoagulation treatment, and can assist doctors in adjusting the dose of anticoagulants when the VTE risk increases.

To summarize, although there are weaknesses of SW-model, including imperfect sensitivity than Caprini and weaker discriminative metrics than Random Forest, the SW-model is more appropriate in surgical patients in Southwestern China than both Caprini and Random Forest. Compared to Caprini, SW-model provided better discriminative capability and simplicity, reducing unnecessary false alarms in clinical applications; to Random Forest, SW-model’s interpretability is essential in guarantee the procedure of risk assessment transparent to clinicians.

### Limitations

This study had several limitations. First, VTE positive cases only included those with a fresh VTE during hospitalization, and did not include VTE occurring after the patient was discharged from the hospital (e.g., in the first 90 days). Compared with previous validation studies of the Caprini model [14], in which VTE was documented until 30 days after surgery, the number of positive cases in the current study may have been underestimated. However, the majority of cases of postoperative VTE occur during the hospitalization; therefore, VTE rarely occurs outside the hospital, and is expected to have little effect on data modeling in this study. Notably, the inclusion of only hospitalized patients makes this model more suitable for risk prediction of VTE in surgical patients.

Second, this was a single-center study. Although 80,946 patients were included in this study, with the data collected for patients who presented between 2019 and 2021, the data was obtained from a single center. Because single center could not represent the population of Chinese surgical patients, multi-center study is needed to validate whether SW-model or its variants is applicable to wider population of Chinese surgical patients.

## Conclusions

Based on statistical analysis of real-world data from surgical patients, the Caprini model was found to overestimate the VTE risk and had insufficient discriminative ability for risk of VTE in surgical patients from Southwestern China. An improved VTE risk assessment model, SW-model, was developed and evaluated against benchmarks, including Caprini and Random Forest. The SW-model contains eight risk factors, reducing the efforts in clinical application and providing superior discriminative capability than the Caprini model. Compared the Random Forest, SW-model’s interpretability is essential in guarantee the procedure of risk assessment transparent to clinicians.

Therefore, the SW-model is more suitable in assessing thrombosis risk in surgical patients in Southwestern China than Caprini and Random Forest. This study paved way for multi-center prospective study on VTE risks of Chinese surgical patients. Should larger scale of studies be conducted in future, Chinese surgical patients could receive more accurate VTE risk assessment; thereby receiving accurate and proper early anticoagulation therapy, which could reduce unnecessary treatments, bleeding risk and economic burdens.

## Supplementary Information

**Additional file 1: Table S1.** Identification rules for new venous thromboembolism (VTE-positive patients) during hospitalization. **Table S2.** Comparison of the characteristics of study participants on training, retrospective and prospective test dataset. **Table S3.** 2005 version of Caprini risk assessment model. **Figure S1.** Feature engineering, model development and evaluation

## Data Availability

The data that support the findings of this study are available from Southwest Hospital but restrictions apply to the availability of these data, which were used under license for the current study, and so are not publicly available. Data are however available from the authors upon reasonable request and with permission of the Ethics Committee of Southwest Hospital.
