# Supplementary material for: Venous thromboembolism risk assessment of surgical patients in Southwest China using real-world data: establishment and evaluation of an improved venous thromboembolism risk model
Source: BMC Med Inform Decis Mak. 2022 Mar 4;22:59. doi: 10.1186/s12911-022-01795-9 (PMC8895056; doi:10.1186/s12911-022-01795-9)

Table S. 1 Identification rules for new venous thromboembolism (VTE-positive patients) during hospitalization.

| **Diagnostic rules** | **Matching objects** | **Matching rules** |
| --- | --- | --- |
| The ICD-10 code of discharge diagnosis contains DVT. | The ICD-10 code of discharge diagnosis. | I80.101, I80.201, I80.204, I80.205, I80.301, I80.303, I81xx02, I82.201, I82.203, I82.302, I82.801, I82.802, I82.804, I82.806, I82.807, I82.808, I82.810, I82.901, I82.902 |
| The ICD-10 code of discharge diagnosis contains PTE. | The ICD-10 code of discharge diagnosis. | Containing 'I26' |
| Findings of the upper or lower extremity blood vessel ultrasound or CT examination suggestive of DVT. | Findings of the upper or lower extremity blood vessel ultrasound or CT examination. | (Possible locations of DVT in the lower extremities) Whether there is thrombosis, embolus, or embolism in the intermuscular vein, anterior tibial vein, posterior tibial vein, popliteal vein, superficial femoral vein, external iliac vein, common iliac vein, iliac vein, femoral vein, gastrocnemius vein, and deep vein  (possible locations of DVT in upper extremities). Whether there is thrombosis, embolus, or embolism in brachial vein, axillary vein, important vein, intermuscular vein, or superficial vein of upper arm.  Cases where the original text denied or showed suspicion of thrombus, embolus, or embolism in the above locations were excluded. |
| Findings of CT angiography of pulmonary artery or lung perfusion scan suggestive of PTE. | Findings of CT angiography of pulmonary artery or lung perfusion scan. | Thrombosis, embolus, or embolism in the pulmonary artery.  Cases where the original text denied or showed suspicion of thrombus, embolus, or embolism in the above locations were excluded. |

Table S. 2 Comparison of the characteristics of study participants on training, retrospective and prospective test dataset

|  | **Training set**  **(2019-2020)** | **Retrospective test set(2019-2020)** | **P value between training and retrospective test set** | **Prospective test set**  **(2021)** | **P value between training and prospective test set** |
| --- | --- | --- | --- | --- | --- |
| Number of visits | 53410 | 13353 |  | 14742 |  |
| Venous thromboembolism | 339  (0.63%) | 85  (0.64%) | 0.981 | 135  (0.92%) | 0.000 |
| Swollen legs | 1082  (2.03%) | 282  (2.11%) | 0.529 | 400  (2.71%) | 0.000 |
| Varicose veins | 808  (1.51%) | 203  (1.52%) | 0.950 | 312  (2.12%) | 0.000 |
| Septicemia | 45  (0.08%) | 8  (0.06%) | 0.374 | 14  (0.09%) | 0.696 |
| Acute myocardial infarction | 475  (0.89%) | 123  (0.92%) | 0.727 | 148  (1.0%) | 0.196 |
| Heart failure | 406  (0.76%) | 102  (0.76%) | 0.965 | 166  (1.13%) | 0.000 |
| History of prior major surgery (<1 month) | 574  (1.07%) | 133  (1.0%) | 0.427 | 158  (1.07%) | 0.976 |
| History of inflammatory bowel disease | 8  (0.01%) | 2  (0.01%) | 1.000 | 0  (0.0%) | 0.288 |
| Serious lung disease | 632  (1.18%) | 170  (1.27%) | 0.394 | 235  (1.59%) | 0.000 |
| Contraception | 53  (0.1%) | 8  (0.06%) | 0.183 | 14  (0.09%) | 0.884 |
| Arthroscopic surgery | 2  (0.0%) | 0  (0.0%) | 0.885 | 0  (0.0%) | 0.835 |
| Malignancy | 15846  (29.67%) | 3867  (28.96%) | 0.109 | 4768  (32.34%) | 0.000 |
| BMI > 25 kg/m2 | 17878  (33.47%) | 4482  (33.57%) | 0.840 | 5233  (35.5%) | 0.000 |
| Laparoscopic surgery | 7391  (13.84%) | 1816  (13.6%) | 0.475 | 2079  (14.1%) | 0.411 |
| Central venous access | 6647  (12.45%) | 1665  (12.47%) | 0.940 | 2334  (15.83%) | 0.000 |
| History of DVT or PE | 86  (0.16%) | 33  (0.25%) | 0.036 | 40  (0.27%) | 0.006 |
| HCY | 1834  (3.43%) | 454  (3.4%) | 0.848 | 629  (4.27%) | 0.000 |
| HIT | 0  (0.0%) | 0  (0.0%) | 0.488 | 0  (0.0%) | 0.520 |
| Family history of VTE | 9  (0.02%) | 2  (0.01%) | 0.880 | 0  (0.0%) | 0.253 |
| Lower extremity arthroplasty | 1  (0.0%) | 1  (0.01%) | 0.327 | 2  (0.01%) | 0.106 |
| Major surgery: > 45minutes | 39463  (73.89%) | 9865  (73.88%) | 0.984 | 11412  (77.41%) | 0.000 |
| Minor surgery: <45 minutes | 13947  (26.11%) | 3488  (26.12%) | 0.984 | 3330  (22.59%) | 0.000 |
| Bedridden duration: 1-72 hours | 2229  (4.17%) | 549  (4.11%) | 0.749 | 876  (5.94%) | 0.000 |
| Bedridden duration: 72+hours | 589  (1.1%) | 157  (1.18%) | 0.473 | 284  (1.93%) | 0.000 |
| Age:18-40 years | 14332  (26.83%) | 3571  (26.74%) | 0.832 | 3571  (24.22%) | 0.000 |
| Age: 41-60 years | 23836  (44.63%) | 5924  (44.36%) | 0.584 | 6902  (46.82%) | 0.000 |
| Age: 61-74 years | 12301  (23.03%) | 3098  (23.2%) | 0.677 | 3379  (22.92%) | 0.778 |
| Age: 75+ years | 2941  (5.51%) | 760  (5.69%) | 0.403 | 890  (6.04%) | 0.013 |
| gender: male | 23412  (43.83%) | 5890  (44.11%) | 0.566 | 6622  (44.92%) | 0.018 |
| gender: female | 29998  (56.17%) | 7463  (55.89%) | 0.567 | 8120  (55.08%) | 0.020 |
| Abnormal platelet counts | 1276  (2.39%) | 311  (2.33%) | 0.684 | 337  (2.29%) | 0.466 |
| Abnormal carcinoembryonic antigen levels | 1065  (1.99%) | 257  (1.92%) | 0.607 | 266  (1.8%) | 0.141 |
| Abnormal triglyceride levels | 1745  (3.27%) | 434  (3.25%) | 0.921 | 617  (4.19%) | 0.000 |
| Abnormal hemoglobin levels | 1832  (3.43%) | 460  (3.44%) | 0.933 | 530  (3.6%) | 0.332 |
| Abnormal D-dimer levels | 4647  (8.7%) | 1142  (8.55%) | 0.586 | 980  (6.65%) | 0.000 |
| History of hypertension | 15016  (28.11%) | 3829  (28.68%) | 0.197 | 3956  (26.83%) | 0.002 |
| History of smoking | 7093  (13.28%) | 1822  (13.64%) | 0.267 | 1934  (13.12%) | 0.609 |
| Endoscopic surgery | 12504  (23.41%) | 3100  (23.22%) | 0.633 | 3546  (24.05%) | 0.103 |
| Intraoperative blood transfusion | 2982  (5.58%) | 733  (5.49%) | 0.672 | 416  (2.82%) | 0.000 |
| Bedridden | 3454  (6.47%) | 891  (6.67%) | 0.388 | 1390  (9.43%) | 0.000 |
| Maximum operation level: 1 | 1964  (3.68%) | 475  (3.56%) | 0.509 | 515  (3.49%) | 0.292 |
| Maximum operation level: 2 | 9438  (17.67%) | 2439  (18.27%) | 0.107 | 2440  (16.55%) | 0.002 |
| Maximum operation level: 3 | 9834  (18.41%) | 2396  (17.94%) | 0.211 | 3145  (21.33%) | 0.000 |
| Maximum operation level: 4 | 11850  (22.19%) | 2886  (21.61%) | 0.154 | 3590  (24.35%) | 0.000 |

Table S. 3 2005 version of Caprini risk assessment model

| Each Risk Factor Represents 1 points | - Age 41-60 years - Minor surgery planned - History of prior major surgery (<1 month) - Varicose veins - History of inflammatory bowel disease - Swollen legs (current) - Obesity (BMI>25) - Acute myocardial infarction - Congestive heart failure (<1 month) - Sepsis lung disease incl. pneumonia (<1 month) - Abnormal pulmonary function (COPD) - Medical patient currently at bed rest - Other risk factors_______ |
| --- | --- |
| Each Risk Factor Represents 2 points | - Age 60-74 years - Arthroscopic surgery - Malignancy (present or previous) - Major surgery (>45 minutes) - Laparoscopic surgery (> 45 minutes) - Patient confined to bed (> 72 hours) - Immobilizing plaster cast (< 1 month) - Central venous access |
| Each Risk Factor Represents 3 points | - Age over 75 years - History of DVT/PE - Family history of thrombosis - Positive Factor V Leiden - Positive Prothrombin 20210A - Elevated serum homocysteine - Positive lupus anticoagulant - Elevated anticardiolipin antibodies - Heparin-induced thrombocytopenia (HIT) - Other congenital or acquired thrombophilia if yes: Type_______ |
| Each Risk Factor Represents 5 points | - Elective major lower extremity arthroplasty - Hip, pelvis or leg fracture (<1 month) - Stroke (<1 month) - Multiple trauma (<1 month) - Acute spinal cord injury (paralysis) (<1 month) |
| For woman only  (Each Risk Factor Represents 1 points) | - Oral contraceptives or hormone replacement therapy - Pregnancy or postpartum (<1 month) - History of unexplained stillborn infant, recurrent spontaneous abortion(≥3), premature birth with toxemia or growth-restricted infant |
| Total Risk Factor Score |  |

Figure S. 1 Feature engineering，model development and evaluation


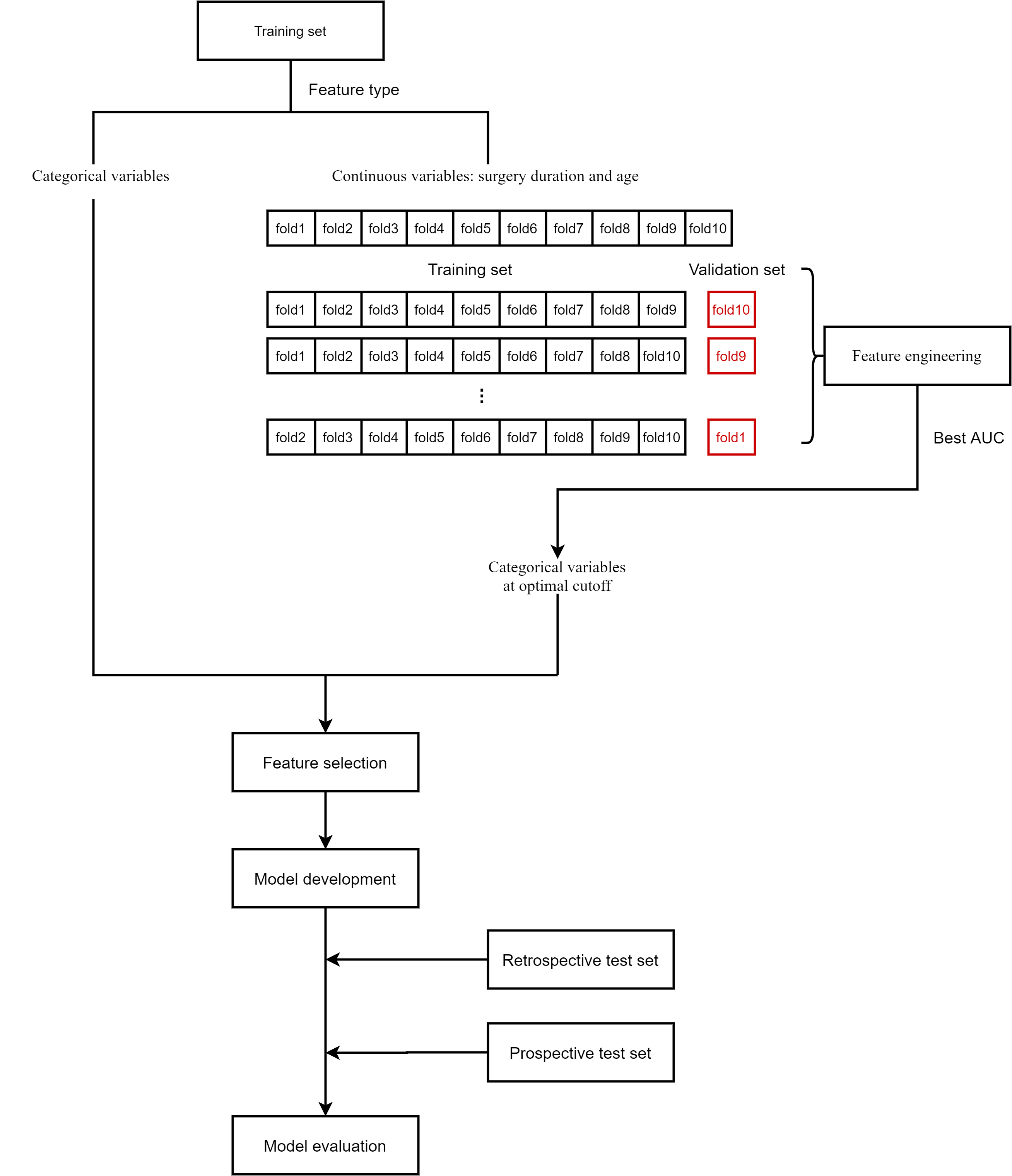

Supplement: Supplementary file 1 — Additional file 1: Table S1. Identification rules for new venous thromboembolism (VTE-positive patients) during hospitalization. Table S2. Comparison of the characteristics of study participants on training, retrospective and prospective test dataset. Table S3. 2005 version of Caprini risk assessment model. Figure S1. Feature engineering, model development and evaluation [file 12911_2022_1795_MOESM1_ESM.doc]
